# Exploring portable NIR spectroscopy in transmittance and reflectance modes for the authentication of Brazilian coffees with geographical indications

**DOI:** 10.1002/jsfa.70493

**Published:** 2026-02-01

**Authors:** Leticia Tessaro, Yhan da Silva Mutz, Mikaela Martins de Bem, Natália de Oliveira Souza, Cleiton Antônio Nunes

**Affiliations:** ^1^ Department of Chemistry Federal University of Lavras Lavras Brazil; ^2^ Department of Food Science Federal University of Lavras Lavras Brazil

**Keywords:** chemometrics, DD‐SIMCA, authentication, spectral fingerprints, coffee traceability

## Abstract

**BACKGROUND:**

Geographical indications (GIs) certify the link between coffee origin and quality, enabling premium pricing and protecting producers. However, the high market value of GI coffees increases their vulnerability to fraud, underscoring the need for reliable and practical authentication methods. Portable near‐infrared (NIR) spectroscopy represents a rapid and non‐destructive alternative, but its capability to discriminate Brazilian GI coffees requires systematic assessment.

**RESULTS:**

This study compared NIR transmittance spectra of aqueous coffee extracts with NIR reflectance spectra of the corresponding ground samples for the authentication of four Brazilian GIs from southeastern Brazil: Cerrado Mineiro, Mogiana Paulista, Mantiqueira de Minas, and Matas de Minas. Distinct spectral signatures were observed in the 900–1650 nm range. Data‐driven soft independent modeling of class analogy (DD‐SIMCA) was employed, resulting in excellent classification performance. Although both acquisition modes showed satisfactory performance during calibration and validation, reflectance consistently outperformed transmittance in the prediction of a test set, achieving accuracies ranging from 97% to 100%. The superior performance of reflectance‐based models was attributed to the preservation of chemically informative features in the solid coffee matrix, including lipids and other compounds poorly extracted into water, whereas aqueous extracts were dominated by water absorption and exhibited greater intraclass variability.

**CONCLUSION:**

Portable NIR spectroscopy, particularly in reflectance mode combined with DD‐SIMCA, provides a fast, non‐destructive, and highly reliable approach for authenticating Brazilian GI coffees. These findings highlight its potential as a practical tool to protect producers and consumers against fraud and to ensure the integrity of products bearing protected geographical indications. © 2026 The Author(s). *Journal of the Science of Food and Agriculture* published by John Wiley & Sons Ltd on behalf of Society of Chemical Industry.

## INTRODUCTION

Coffee is the world's second most consumed beverage and one of the most traded commodities. Brazil is the leading producer, accounting for approximately 38% of global coffee production in the 2024/2025 harvest.^1^ Coffee cultivation in Brazil covers about 2.2 million hectares and involves nearly 300 000 producers, concentrated mainly in the states of Minas Gerais, Espírito Santo, and São Paulo.^2^

Demand for high‐quality coffee has increased in recent years, driven by its distinctive sensory attributes and consumer interest in novel coffee experiences ^3^. Consumers are often willing to pay premium prices for products originating from regions with protected geographical indications (GIs), as these coffees exhibit qualities and reputations that arise essentially or exclusively from their specific geographical environment.^4^

In Brazil, the National Institute of Industrial Property is responsible for registering GIs for coffee under either a Designation of Origin (DO) or an Indication of Provenance (IP), ensuring product verification through an official seal. This certification assures producers of the quality associated with GI coffee and protects these products against fraud by preventing imitations from neighboring regions that might otherwise claim association with the same GI.^5^ Currently, Brazil has a diverse portfolio of coffee GIs, with 14 products officially registered by the Brazilian Ministry of Agriculture, Livestock, and Food Supply.^6^

Coffee quality and GI attribution are currently assessed predominantly through cupping sessions conducted by trained tasters. Consequently, rapid and objective analytical methods are required to support quality differentiation and authenticity verification.^3^ Several analytical techniques have been applied to determine coffee origin, including chromatographic analysis,^7^ nuclear magnetic resonance,^8^ and electrochemical sensors.^3^ However, these approaches are often costly, require extensive sample preparation and long analysis times, and generate considerable chemical waste.

Near‐infrared spectroscopy (NIRS) represents a promising alternative to overcome these limitations. This technique requires minimal or no sample preparation and preserves sample integrity.^9^ NIR spectra can be acquired in transmittance and reflectance modes, broadening the applicability of the technique to different matrices and physical states.^10^ The increasing availability of portable NIR devices has further enhanced its practical relevance by enabling non‐destructive, *in situ* analysis with reduced cost, robustness, and ease of use, making it particularly attractive for routine characterization and on‐site decision‐making, which are essential for detecting coffee fraud.^11^, ^12^

In a previous study, Tessaro *et al*. (2025) demonstrated that coffees with protected GIs from southeastern Brazil could be discriminated based on the profiles of bioactive compounds in aqueous extracts, as determined by high‐performance liquid chromatographic (HPLC) analysis.^13^ In this context, the present study aimed to evaluate whether aqueous coffee extracts analyzed by NIR transmittance concentrate more useful spectral information than the corresponding ground coffee samples analyzed by NIR reflectance for the authentication of their GIs. For this purpose, coffees from four regions in southeastern Brazil with certified GIs were considered, and authentication models were developed using data‐driven soft independent modeling of class analogy (DD‐SIMCA).

## METHODOLOGY

### Sampling

**Figure 1:**
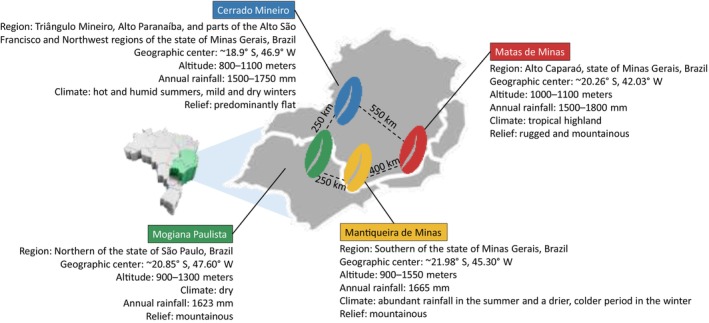
Geographic regions in southeastern Brazil: Cerrado Mineiro (CM), Mogiana Paulista (MG), Mantiqueira de Minas (MQ), and Matas de Minas (MM). The distances between the regions are approximate and were estimated based on their geographic centers.

Twenty coffee samples from each region under study were purchased from local markets in Minas Gerais, Brazil. All samples belonged to the species *Coffea arabica* and represented distinct production lots. The selected regions are located in southeastern Brazil (Fig. 1): Cerrado Mineiro (CM), Mogiana Paulista (MG), Mantiqueira de Minas (MQ), and Matas de Minas (MM). To ensure comparability among regions and product quality, only coffees bearing the GI seal granted by the National Institute of Industrial Property were included. An additional external class (EXT), composed of eight specialty coffee samples from southeastern Brazil without GI certification, was incorporated for model testing.

### Sample preparation and analysis

All coffee samples were originally acquired at a medium roast level. To ensure uniformity, the samples were ground to a medium grind size for 30 s using a Hamilton Beach grinder (model 80 393‐BZ127, Hamilton Beach Brands, Inc., Richmond, VA, USA) prior to NIRS analysis. Each sample was analyzed using two independent aliquots, each measured in triplicate by NIRS, and the resulting spectra were averaged.

Analyses were performed using two portable NIR devices. The first device, an NIR‐S‐G1 (InnoSpectra, Hsinchu, Taiwan), acquires spectral data in the near‐infrared range (900–1650 nm) in reflectance mode (NIR‐R). For these measurements, no sample preparation was required, and spectra were collected directly from ground coffee placed on a porcelain plate. The second device, an InnoSpectra NIR‐S‐T2, acquires spectral data in the same wavelength range in transmittance mode (NIR‐T). For this analysis, a coffee extract was prepared by placing 1.0 g of roasted and ground coffee into qualitative‐grade filter paper (80 g; Unifil AG Filtertechnik, Niederlenz, Switzerland) and extracting it with 10 mL distilled water at 90 °C. The extract was then cooled to 25 °C, transferred to a quartz cuvette, and analyzed. Spectra were recorded as absorbance values at 4 nm intervals in both acquisition modes.

### Chemometric analyses

Synthetic samples were generated using the synthetic minority oversampling technique (SMOTE),^14^ to improve the robustness of class modeling and parameter optimization, particularly given the limited number of commercially available coffee samples bearing a GI seal. SMOTE was applied using five nearest neighbors to generate 20 synthetic samples for each coffee class (CM, MG, MQ, and MM), resulting in 40 samples per class (20 original and 20 synthetic). In addition, eight samples from the external class were included. Therefore, the final dataset comprised 168 samples measured across 211 wavelengths.

The dataset was divided into training, validation, and test sets. The training set consisted of 20 samples of the target class (15 original and 5 synthetic). The validation set included 19 samples, comprising 10 synthetic samples of the target class and 3 original samples from each non‐target class. The test set consisted of 69 samples, including 10 samples of the target class (5 original and 5 synthetic), 17 original samples from each non‐target class, and 8 external samples. Samples were randomly assigned to each set.

Principal component analysis (PCA) was performed to evaluate the natural clustering of GI coffees using spectra acquired in both reflectance and transmittance modes^15^. Prior to PCA, the spectra were preprocessed using a first derivative calculated with a first‐degree polynomial and a 15‐point window.

For authentication modeling, the one‐class classifier DD‐SIMCA was employed. Model development followed the guidelines proposed by Kucheryavskiy *et al*.^16^ Models were trained using the training set, and parameter optimization was performed by maximizing prediction efficiency in the validation set. As each GI class was modeled individually, the optimal preprocessing strategy and number of principal components (PCs) were independently evaluated for each class. The effectiveness of different spectral preprocessing methods, including standard normal variate (SNV) and Savitzky–Golay derivatives, was evaluated, with various combinations of window size, polynomial order, and derivative order tested for the Savitzky–Golay filter. The final model for each class was selected based on the preprocessing approach that maximized validation performance. Model performance was subsequently evaluated using the independent test set. All models were developed using a significance level of *α* = 5% and *γ* = 1%, operating in classical mode.

Model performance was assessed using sensitivity (SEN), specificity (SPE), efficiency (EFI), accuracy (ACU), and F1‐score (F1‐S), as defined in Eqns (1)–(5):

(1) $$\mathrm{SEN}=\frac{\mathrm{TP}}{\mathrm{TP}+\mathrm{FN}}$$

(2) $$\mathrm{SPE}=\frac{\mathrm{TN}}{\mathrm{TN}+\mathrm{FP}}$$

(3) $$\mathrm{EFI}=\sqrt{\mathrm{SEN}\;\times \;\mathrm{SPE}}$$

(4) $$\mathrm{ACU}=\frac{\mathrm{TP}+\mathrm{TN}}{\mathrm{TP}+\mathrm{TN}+\mathrm{FP}+\mathrm{FN}}$$

(5) $$F1-S=\frac{2\;\times \;\mathrm{TP}\;}{2\;\times \;\mathrm{TP}+\mathrm{FP}+\mathrm{FN}}$$

where TP, TN, FP, and FN represent the numbers of true positives, true negatives, false positives, and false negatives, respectively. These metrics were calculated for each target class against the non‐target classes. Sensitivity reflects the model's ability to correctly accept target‐class samples, whereas specificity quantifies its capacity to correctly reject non‐target samples. Efficiency provides an integrated measure of performance by combining sensitivity and specificity. Accuracy represents the proportion of correct predictions, and F1‐score summarizes the balance between false positives and false negatives.

All chemometric analyses were performed using the freely available web application mda.tools.^16^

## RESULTS AND DISCUSSION

### Spectral analysis

**Figure 2:**
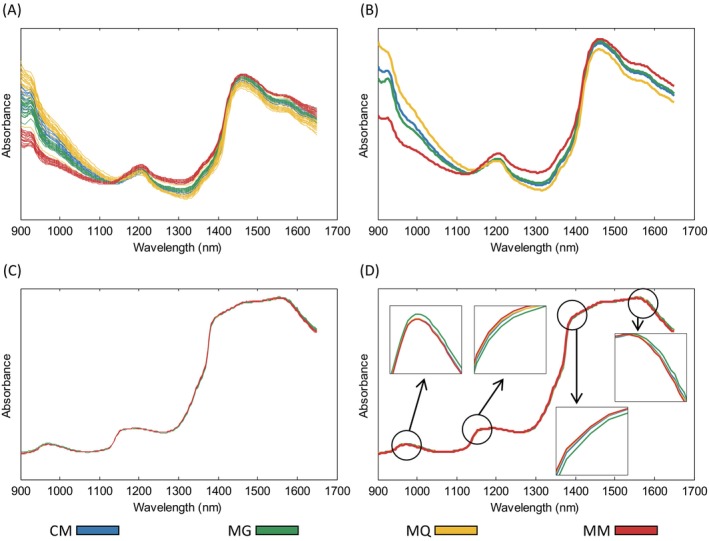
Near‐infrared spectra acquired in reflectance (A) and transmittance (C) for coffee samples from Cerrado Mineiro (CM), Mogiana Paulista (MG), Mantiqueira de Minas (MQ), and Matas de Minas (MM), with the corresponding class mean spectra shown in (B) and (D). Original and synthetic spectra are included.

The NIR spectra of the coffee samples exhibited distinct fingerprints depending on the acquisition mode (Fig. 2). Although both devices operated within the same wavelength range (900–1650 nm), they generated markedly different spectral profiles. These differences are mainly associated with nonlinear effects caused by light scattering. In NIR‐R, ground coffee samples were analyzed directly, and diffuse reflectance was measured.^17^ Diffuse reflectance carries information on both the chemical composition and the physical microstructure of the sample, with particle size, particle size distribution, porosity, surface roughness, and sample compaction strongly influencing the effective optical path length. These factors give rise to baseline shifts, multiplicative effects, and other nonlinear spectral distortions.^17^ In NIR‐T, a filtered coffee extract was analyzed, minimizing the presence of particulate material and scattering effects. The transmitted light travels along a more controlled optical path, resulting in spectra with greater baseline stability and fewer multiplicative effects compared to NIR‐R. Consequently, NIR‐T spectra are less affected by scattering‐induced nonlinearities, whereas NIR‐R spectra more strongly reflect combined chemical and physical variability.

NIR spectroscopy is based on molecular vibrational transitions, giving rise to overtone and combination bands. Within the NIR region, higher‐order overtones occur mainly below 900 nm and typically present low‐intensity absorption features, while second overtones are observed approximately between 900 and 1400 nm, and first overtones dominate the region from about 1400 to 2000 nm, often overlapping with combination bands at higher wavelengths.^18^ Coffee exhibits a chemically complex matrix, with more than 800 compounds identified to date. Nevertheless, it is a well‐characterized material, and its main constituents, such as lipids, carbohydrates, proteins, caffeine, and phenolic compounds, contribute systematically to the NIR spectra through their characteristic vibrational features.^18^

The NIR reflectance spectra (Fig. 2(A),(B)) revealed clear spectral differences among classes. The absorption feature observed around 950 nm can be broadly associated with second‐overtone contributions of O—H and C—H stretching vibrations, which are common in organic matrices and reflect the presence of compounds such as lipids, carbohydrates, proteins, and phenolic constituents.^18^, ^19^ The band near 1200 nm is mainly related to second‐overtone C—H stretching vibrations, commonly observed in lipids and carbohydrates and also influenced by other organic components present in coffee, including caffeine.^9^, ^18^ The absorption band typically observed around 1400–1450 nm, attributed to the first overtone of O—H stretching from water, appears with reduced intensity in the spectra of roasted and ground coffee due to the relatively low moisture content of the samples. The region between 1500 and 1600 nm is dominated by first‐overtone N—H stretching and overlapping combination bands of O—H and C—H, reflecting contributions from nitrogen‐containing compounds and phenolic structures present in coffee.^9^, ^18^

The NIR spectra of the coffee extracts (Fig. 2C) were largely dominated by the strong absorption features of water, resulting in spectra that closely resemble those of pure water. Nevertheless, subtle but systematic differences among classes were observed, particularly in the regions around 950, 1150, 1400, and 1550 nm (Fig. 2(D)). These spectral regions are sensitive to water structure and hydrogen‐bonding interactions, as well as to overtone and combination bands associated with O—H and C—H functional groups, which may indirectly reflect chemically relevant information related to dissolved organic compounds in the coffee extracts.^18^

Examination of the mean NIR‐R spectra for each class (Fig. 2(B)) suggests a clear natural separation among classes, supporting the development of robust classification models. In contrast, this assumption cannot be confidently extended to the mean NIR‐T spectra, as the classes show substantial spectral overlap and lack distinct peak differences, despite the presence of subtle variations (Fig. 2(D)).

### Exploratory analysis

**Figure 3:**
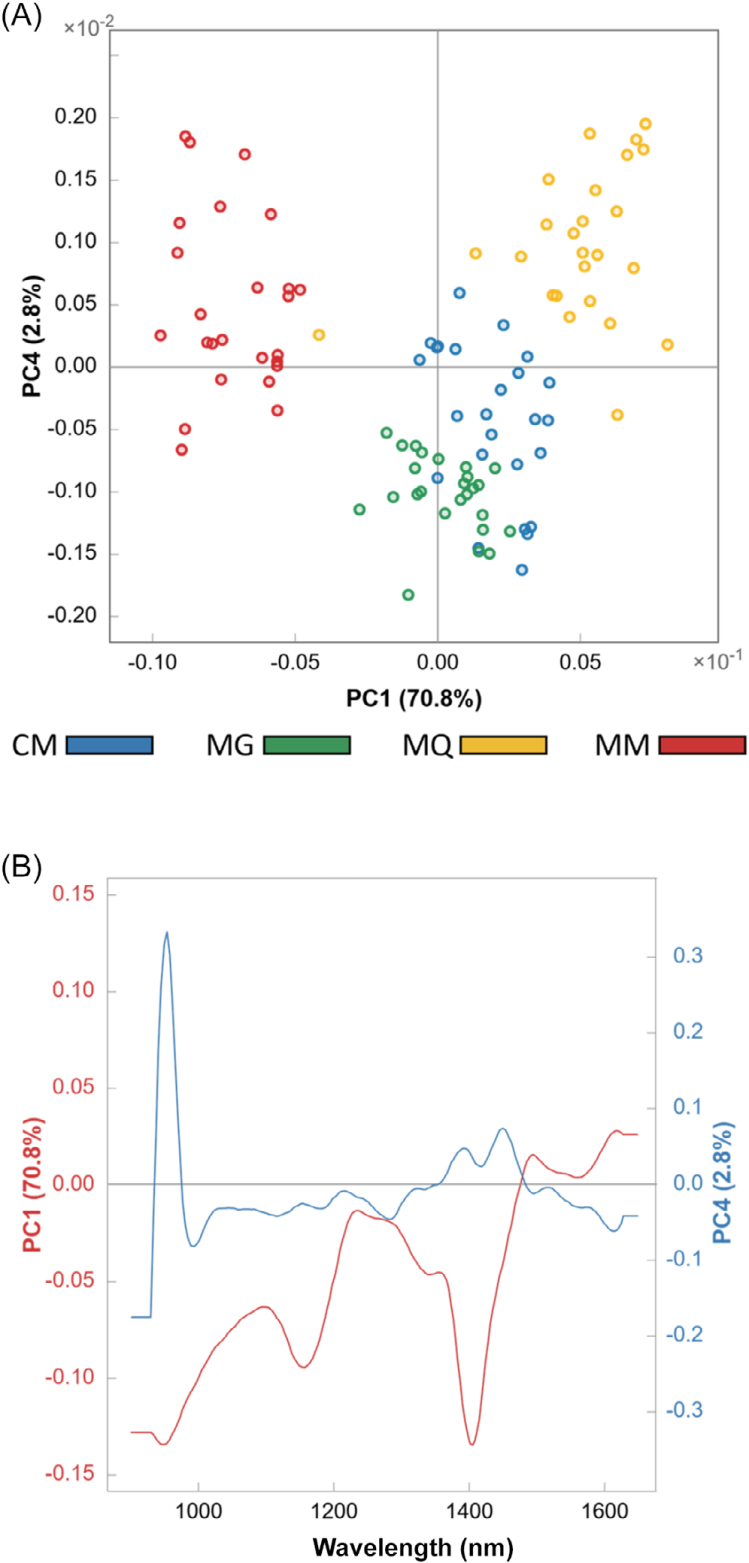
Principal component analysis scores (A) and loadings (B) plots obtained from the near‐infrared spectra in reflectance mode of the four coffee samples: CM, Cerrado Mineiro; MG, Mogiana Paulista; MQ, Mantiqueira de Minas; MM, Matas de Minas.

**Figure 4:**
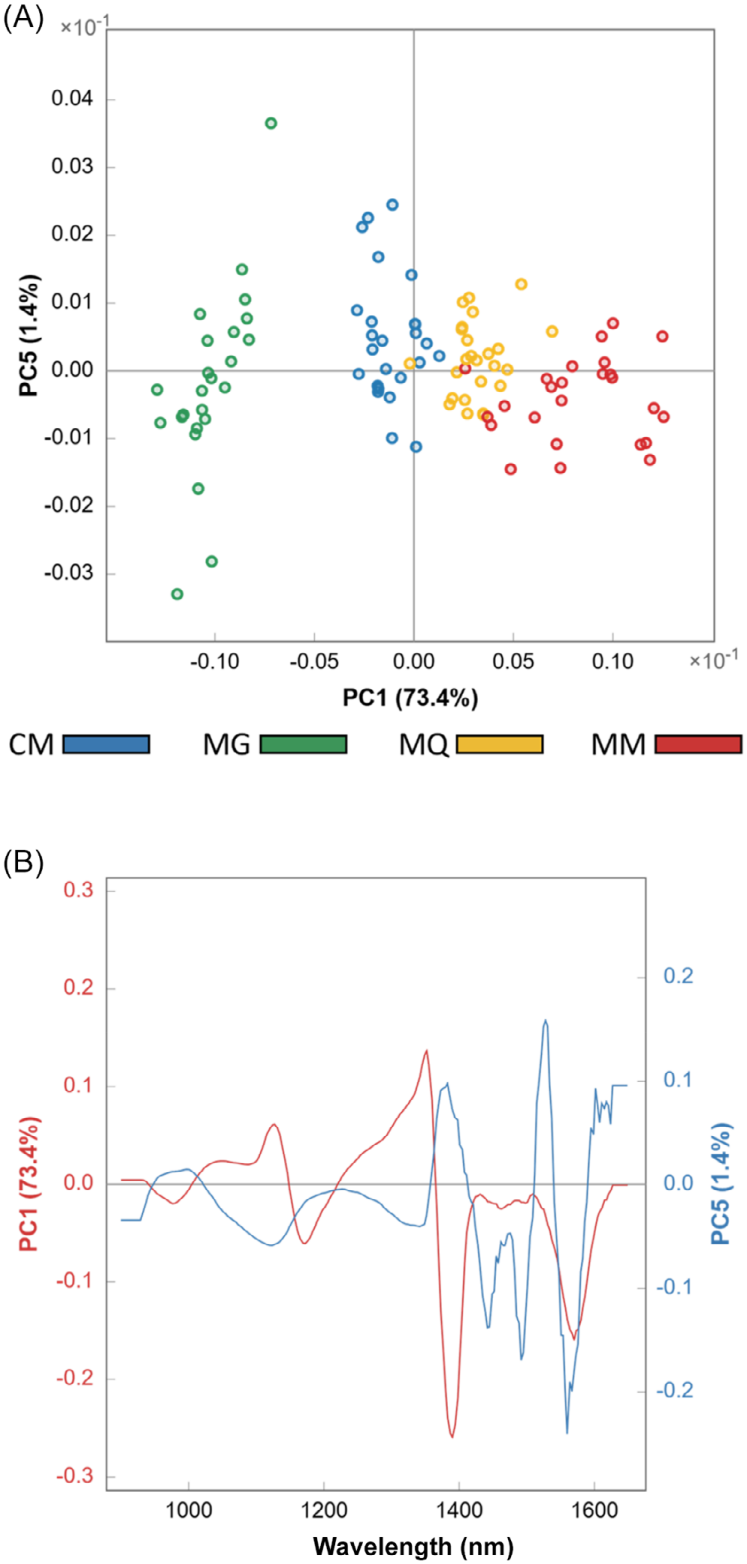
Principal component analysis scores (A) and loadings (B) plots obtained from the near‐infrared spectra in transmittance mode of the four coffee classes: CM, Cerrado Mineiro; MG, Mogiana Paulista; MQ, Mantiqueira de Minas; MM, Matas de Minas.

An exploratory analysis was first performed to assess the natural clustering of the coffee classes. PCA was applied separately to the two spectral datasets (NIR‐R and NIR‐T), and the results were examined using scores and loadings plots (Figs 3 and 4).

A clear separation among the coffee classes based on NIR‐R was achieved using PC1 and PC4, which together explained 73.6% of the total variance (Fig. 3(A)). The MM and MQ classes exhibited distinct natural clustering, showing negative and positive correlations with PC1, respectively, without overlap with the remaining classes. Clustering was also observed for the CM and MG classes, although with only minor overlap. Examination of the PC1 loadings (Fig. 3(B)), which accounted for 70.8% of the variance, revealed strong contributions in the regions around 900–1000 , 1170, and 1400 nm. In contrast, PC4 showed a pronounced contribution near 950 nm and a moderate contribution around 1450 nm.

A reasonable separation of the coffee classes based on NIR‐T was achieved using PC1 and PC5, which together explained 74.8% of the variance (Fig. 4(A)). The scores plot indicated that the MQ and MM classes were positively correlated with PC1, whereas the CM and MG classes were predominantly located in the negative region of PC1. The MG class was clearly separated from the other classes, while the remaining classes, although not fully resolved, exhibited natural grouping tendencies. The loadings plot (Fig. 4(B)) showed that PC1 and PC5 presented pronounced contributions around 950, 1150, 1400, and 1550 nm, consistent with the spectral regions highlighted in the extract spectra (Fig. 2(D)), where subtle but systematic differences among classes were observed.

The interpretation of NIR transmittance spectra of aqueous systems is often approached through the aquaphotomics framework,^20^ in which water is regarded as an active spectroscopic probe whose absorption features reflect changes in its hydrogen‐bonding network induced by solutes. While this conceptual approach is well established and particularly effective for concentrated or tightly controlled systems, its application to the present study requires caution. Aquaphotomic analyses typically rely on very small variations in water absorption bands, often reported on the order of 10^−4^ absorbance units relative to pure water, which approach the practical signal‐to‐noise limits of conventional NIR spectrophotometers.^20^ Such subtle variations are highly sensitive to instrumental drift, temperature fluctuations, and sample handling.

Although water–solute interactions are inherently present in aqueous coffee extracts, the available instrumental sensitivity and experimental conditions indicate that aquaphotomic effects are unlikely to represent the dominant source of the observed class discrimination. Instead, the spectral differences are more plausibly dominated by direct absorption contributions from the extracted compounds, with water acting primarily as a solvent matrix. Coffee extracts constitute chemically complex solutions containing organic acids, phenolic compounds, and other bioactive molecules^13^, ^21^ Consequently, the discrimination observed among classes most likely reflects genuine compositional differences in the extracts rather than amplified perturbations of the water hydrogen‐bonding structure alone. These results indicate that the extraction process yields chemically distinct solutions whose differences are analytically significant.

In both acquisition modes, the first principal component, which explained the largest proportion of variance, retained the most relevant information for GI discrimination. Nevertheless, additional information captured by lower‐variance components also proved to be important. GI regions differ through subtle chemical variations that contribute to specific sensory attributes, such as aroma and flavor. Accordingly, it is reasonable that principal components explaining smaller portions of the variance contribute to class differentiation. Although discrimination of coffees based on GI is a complex and labor‐intensive task, previous studies have demonstrated that differences in the levels of bioactive compounds, including caffeine, chlorogenic acids, trigonelline, and theobromine, can effectively distinguish GI classes.^13^, ^22^

The results of the present study are consistent with findings reported in the literature. Pimenta *et al*. characterized the geographical origin of Brazilian coffees using ultra‐high‐performance liquid chromatography coupled with high‐resolution mass spectrometry and identified carbohydrates, amino acids, organic acids, polyphenols, alkaloids, and lipids as the main contributors to class separation.^21^ In that study, the primary biomarker compounds used to differentiate GI classes were ferulic acid and chlorogenic acids (4,5‐caffeoylquinic acid and 3,5‐caffeoylquinic acid), with coffees from the Cerrado Mineiro region exhibiting elevated concentrations of these markers. Furthermore, Tessaro *et al*. (2025) identified potential biomarker compounds for each GI class considered in the present study using HPLC coupled with diode array detection.^13^ The MQ class showed the highest concentrations of ferulic acid, the CM class exhibited lower concentrations, and the MG and MM classes showed no detectable levels of this compound. In addition, *p*‐coumaric acid was identified as a potential biomarker, with the highest concentrations observed in the MG class, lower concentrations in the CM class, and an absence in the MQ and MM classes.

Based on the exploratory analysis, the spectral signals constituted distinctive fingerprints that enabled the separation and clustering of coffee samples according to their GIs. A comparison between the two acquisition modes indicated that NIR reflectance provided improved class separation, with only minor overlap observed between the CM and MG classes.

### Supervised classification

The objective of this study was to authenticate each GI coffee using NIR data; therefore, the DD‐SIMCA approach was employed. Authentication is defined as the process of determining whether an object is indeed what it is claimed to be.^23^ Accordingly, a one‐class classification strategy is required. DD‐SIMCA, which is based on PCA, is trained exclusively using samples from the target class. The literature consistently emphasizes that discriminant analysis is unsuitable for authentication purposes, as the development of discriminant models requires all classes to be known and well defined in advance.^23^, ^24^ In fraud‐related scenarios, particularly in the coffee sector, it is unrealistic to anticipate all potential adulteration strategies. Consequently, DD‐SIMCA represents an appropriate tool for authentication, as each class is modeled independently and samples not belonging to the target class can be effectively detected.^16^

For the NIR‐R models, the Savitzky–Golay derivative was identified as the most effective preprocessing method for all classes, except for the MQ class, which achieved optimal performance SNV preprocessing (Table 1). All models reached 100% sensitivity during calibration and exhibited perfect classification in the validation step. No evidence of overfitting was observed, as supported by the main figures of merit and by the agreement between the expected and observed numbers of extremes in the validation set.^16^

**Table 1:** Model performance metrics for differentiating GI coffees using data‐driven soft independent modeling of class analogy based on near‐infrared spectroscopy in reflectance mode

Class		CM	MG	MQ	MM
# of PC		5	3	4	4
Pretreatment		SG (1, 2, 7)	SG (1, 2, 11)	SNV	SG (1, 2, 7)
Calibration	SEN	100.0	100.0	100.0	100.0
Validation	SEN	100.0	100.0	100.0	100.0
	SPE	100.0	100.0	100.0	100.0
	EFI	100.0	100.0	100.0	100.0
	ACU	100.0	100.0	100.0	100.0
	F1‐S	100.0	100.0	100.0	100.0
Test	SEN	100.0	100.0	90.0	90.0
	SPE	100.0	96.6	100.0	100.0
	EFI	100.0	98.3	94.9	94.9
	ACU	100.0	97.1	98.6	98.6
	F1‐S	100.0	90.9	94.7	94.7

Abbreviations: ACU, accuracy; CM, Cerrado Mineiro; EFI, fficiency; F1S, F1‐score; MG, Mogiana Paulista; MM, Matas de Minas; MQ, Mantiqueira de Minas; PC, principal components; SEN, sensitivity; SG, Savitzky–Golay filter (window size, polynomial order, derivative order); SNV, standard normal variate; SPE, specificity.

The optimal number of PCs varied among the models, with five PCs required for the CM class, four PCs for the MQ and MM classes, and three PCs for the MG class. In SIMCA‐based modeling, a higher number of PCs may reflect increased class complexity, which can be associated with greater heterogeneity or uncontrolled variability within the class. Such behavior is expected when analyzing a diverse set of commercial coffee samples from different brands and production lots, as was the case in the present study.

**Figure 5:**
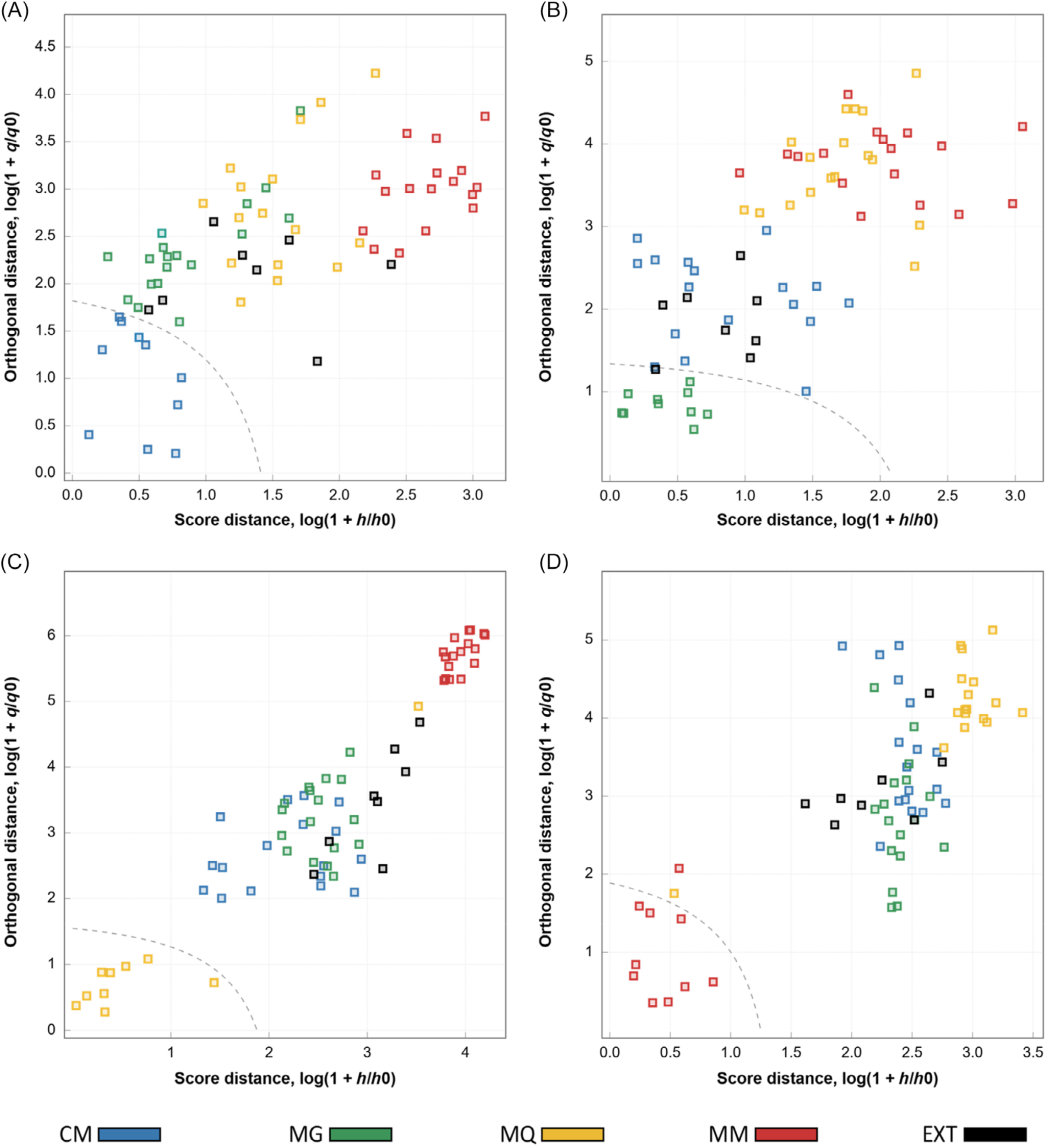
Acceptance plots for differentiating GI coffees from the test set using data‐driven soft independent modeling of class analogy based on near‐infrared spectroscopy in reflectance mode. (A) CM (Cerrado Mineiro); (B) MG (Mogiana Paulista); (C) MQ (Mantiqueira de Minas); and (D) MM (Matas de Minas).

The optimized models were subsequently applied to the test set, which included target samples, non‐target samples, and an external class without GI certification. High accuracy values were obtained for all classes (Table 1), indicating the robustness of the decision thresholds established for each region. According to the acceptance plots (Fig. 5) and the confusion matrix (Table 2), only a limited number of misclassifications occurred, including one CM and one external sample classified as MG, one MQ sample classified as not‐MQ, and one MM sample classified as not‐MM. As a result, the CM, MQ, and MM models achieved 100% specificity, while the MG model exhibited a specificity of 96.6%, demonstrating a strong ability to correctly reject non‐target samples. High recognition capability for target‐class samples was also observed, with the CM and MG models achieving 100% sensitivity, whereas the MQ and MM models showed sensitivity values of 90%.

**Table 2:** Confusion matrix for differentiating GI coffees from the test set using data‐driven soft independent modeling of class analogy based on near‐infrared spectroscopy in reflectance mode

		Actual				
		CM	MG	MQ	MM	EXT
Predicted	CM	10	0	0	0	0
	not‐CM	0	17	17	17	8
	MG	1	10	0	0	1
	not‐MG	16	0	17	17	7
	MQ	0	0	9	0	0
	not‐MQ	17	17	1	17	8
	MM	0	0	0	9	0
	not‐MM	17	17	17	1	8

Abbreviations: CM, Cerrado Mineiro; MG, Mogiana Paulista; MQ, Mantiqueira de Minas; MM, Matas de Minas; EXT, external class.

For class modeling based on NIR‐T, SNV was identified as the most effective preprocessing method for all classes except MG, which performed better when using the Savitzky–Golay derivative (Table 3). Overall, model performance in transmittance mode was inferior to that obtained using reflectance data. All models achieved 100% sensitivity during both calibration and validation; however, perfect classification was observed only for the MG class. Nevertheless, all NIR‐T models achieved accuracies of at least 90%, indicating a satisfactory ability to discriminate between target and non‐target samples.

**Table 3:** Model performance metrics for differentiating GI coffees using data‐driven soft independent modeling of class analogy based on near‐infrared spectrocopy in transmittance mode

Class		CM	MG	MQ	MM
# of PC		2	3	4	2
Pretreatment		SNV	SG (1, 11)	SNV	SNV
Calibration	SEN	100.0	100.0	100.0	100.0
Validation	SEN	100.0	100.0	100.0	100.0
	SPE	88.9	100.0	88.9	88.9
	EFI	94.3	100.0	94.3	89.4
	ACU	94.7	100.0	94.7	89.5
	F1‐S	95.2	100.0	95.2	90.0
Test	SEN	100.0	100.0	90.0	80.0
	SPE	100.0	91.5	94.9	98.3
	EFI	100.0	95.7	92.4	88.7
	ACU	100.0	92.8	94.2	95.7
	F1‐S	100.0	80.0	81.8	84.2

Abbreviations: ACU, accuracy; CM, Cerrado Mineiro; EFI, efficiency; F1S, F1‐score; MG, Mogiana Paulista; MM, Matas de Minas; MQ, Mantiqueira de Minas; PC, principal components; SEN, sensitivity; SG, Savitzky–Golay filter (window size, polynomial order, derivative order); SNV, standard normal variate; SPE, specificity.

**Figure 6:**
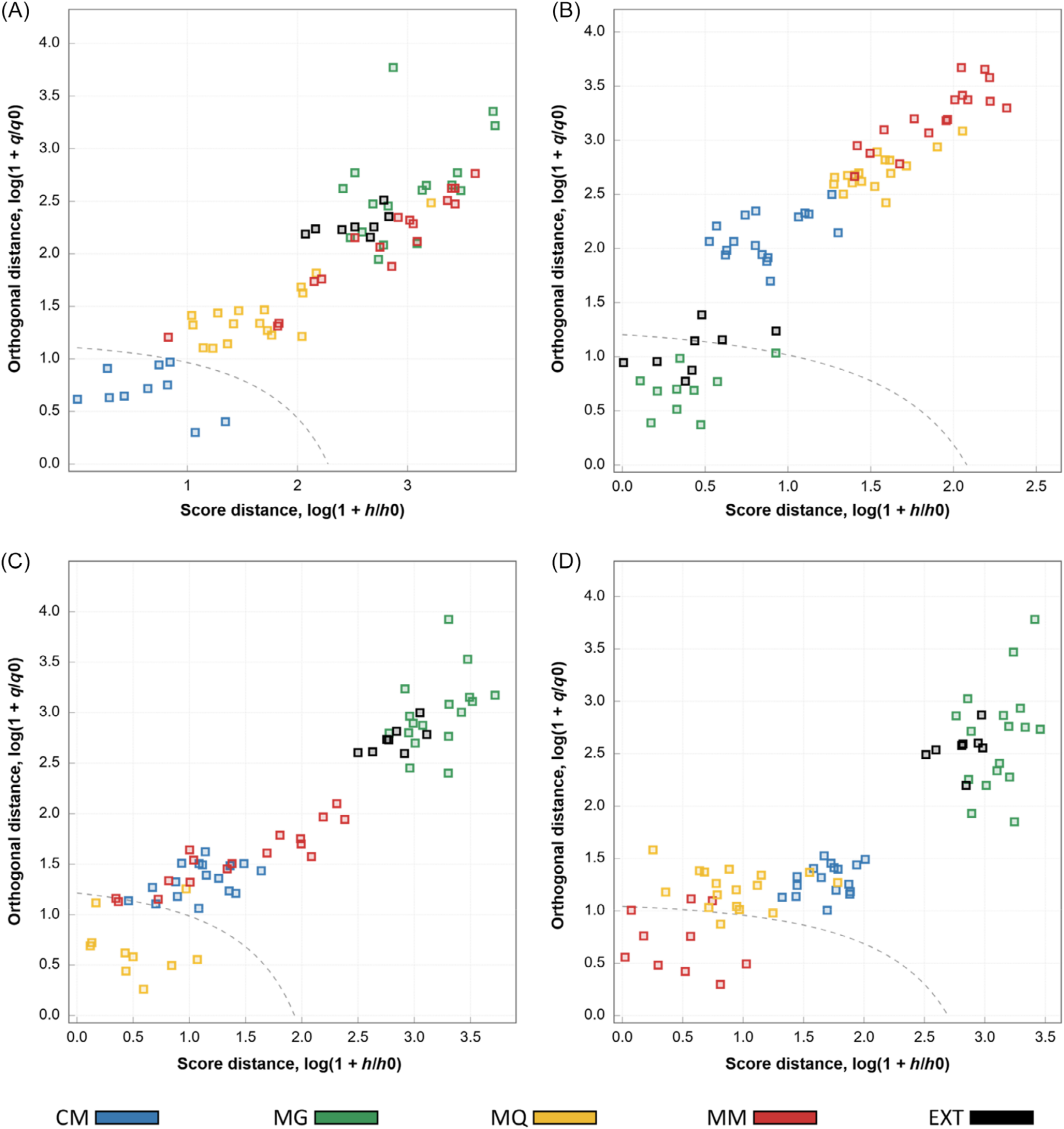
Acceptance plots for differentiating GI coffees from the test set using data‐driven soft independent modeling of class analogy based on near‐infrared spectroscopy in transmittance mode. (A) CM (Cerrado Mineiro); (B) MG (Mogiana Paulista); (C) MQ (Mantiqueira de Minas); and (D) MM (Matas de Minas).

In the test set, perfect classification was obtained only for the CM class. Although the MG model achieved 100% sensitivity by correctly recognizing all target samples, false positives were observed, leading to reduced specificity. Based on the PCA scores plot derived from the NIR‐T data (Fig. 4(A)), perfect classification for the MG class might have been anticipated. However, the acceptance plots (Fig. 6) and the confusion matrix (Table 4) revealed that the false positives originated primarily from misclassification of the external class, which was not included in the exploratory analysis restricted to GI coffees. Indeed, the acceptance plots indicated that the MG class exhibited greater similarity to the external class, while remaining clearly separated from the other GI classes across all models. This suggests that the aqueous extracts of the external samples were chemically similar to those of the MG samples, thereby increasing the difficulty of discrimination. For the MQ and MM classes, misclassifications mainly involved samples from these classes and, to a lesser extent, samples from the CM class (Fig. 6; Table 4), which is consistent with the minor overlaps observed in the PCA scores plot (Fig. 4(A)).

**Table 4:** Confusion matrix for differentiating GI coffees from the test set using data‐driven soft independent modeling of class analogy based on near‐infrared spectroscopy in transmittance mode

		Actual				
		CM	MG	MQ	MM	EXT
Predicted	CM	10	0	0	0	0
	not‐CM	0	17	17	17	8
	MG	0	10	0	0	5
	not‐MG	17	0	17	17	3
	MQ	1	0	9	2	0
	not‐MQ	16	17	1	15	8
	MM	0	0	1	8	0
	not‐MM	17	17	16	2	8

Abbreviations: CM, Cerrado Mineiro; EXT, external class; MG, Mogiana Paulista; MM, Matas de Minas; MQ, Mantiqueira de Minas.

It is important to emphasize that the authentication models were challenged using an external class composed of specialty coffee samples from southeastern Brazil without GI certification. This represents a particularly demanding scenario, as these samples originate from cultivation areas geographically close to the GI regions investigated. Moreover, discriminating among different classes of specialty coffees is inherently more complex than distinguishing specialty coffees from non‐specialty samples. Even under these challenging conditions, the DD‐SIMCA models based on portable NIR spectroscopy demonstrated satisfactory performance, achieving high sensitivity and specificity with respect to the external class, particularly when reflectance mode was employed.

### Performance of the NIR approaches compared to other analytical techniques

Numerous studies have addressed the geographic authentication of coffee, employing a broad range of analytical techniques, as no standardized methodology has yet been established for this purpose (Table 5). The literature consistently demonstrates that, when combined with appropriate chemometric approaches, these techniques achieve high classification performance, typically exceeding 90%. These findings highlight the strong potential of analytical authentication strategies, particularly in light of growing concerns regarding coffee fraud driven by the continued expansion of the global market.

**Table 5:** Summary of studies in the literature employing different analytical techniques and chemometric tools for the GI authentication of coffee (*Coffea arabica*)

Instrument	Mode	Technique	Goal	Accuracy (%)	Reference
ICP‐OES	NA	MLP‐ANN	Characterization of four regions from Mexico	93–98	^25^
Screen printed electrodes (Gpt‐PLA)	NA	SIMCA	Differentiation of Arabica coffee from three regions of GI	92	^6^
NIRS with Integrating Sphere	Interactance	SIMCA	Authentication of four Arabica coffees from Indonesia	NR	^26^
NIRS	Reflectance	SVM	Classification of four locations from Paraná (Brazil) in green arabica coffee	100	^9^
FTIR	Reflectance			95–98	
UV–visible	Absorption	DD‐SIMCA	Authentication of green coffee beans from Cerrado Mineiro (Brazil)	93	^27^
NIR portable	Reflectance	DD‐SIMCA	Authentication of four regions from Brazil	97–100	This work

Abbreviations: DD‐SIMCA, data‐driven soft independent modeling of class analogy; FTIR, Fourier transform infrared spectroscopy; NIRS, near‐infrared spectroscopy; Gpt‐PLA, electrodes printed on graphene–polylactic acid composite; ICP‐OES, inductively coupled plasma–optical emission spectroscopy; MLP‐ANN, multilayer perceptron–artificial neural network; NA, not applicable; NR, not reported; SVM, support vector machine; UV–visible, ultraviolet–visible spectroscopy.

The results obtained using portable NIR spectroscopy are particularly noteworthy, as they reveal classification performance comparable to that reported for benchtop instruments, such as those employed by Bona *et al*., with 100% accuracy achieved in both cases.^9^ While the studies by Bona *et al*. and dos Santos *et al*.^27^ relied on fixed laboratory instrumentation, thereby limiting in‐field applicability, similarly high performance was achieved here using portable devices. Moreover, in contrast to high‐cost techniques reported in the literature, such as inductively coupled plasma optical emission spectrometry (ICP‐OES),^25^ which require labor‐intensive sample preparation, portable NIR spectroscopy enables rapid, non‐destructive, and on‐site analysis.

Furthermore, to the best of our knowledge, no previous studies have reported a direct comparison between portable NIR spectroscopy operated in reflectance and transmittance modes for the authentication of coffee geographical indications. Accordingly, the present study provides the first systematic comparison of these two acquisition modes using portable NIR devices for the GI authentication of Brazilian coffees, highlighting a fast, cost‐effective, and operationally simple analytical alternative suitable for in‐field applications.

## CONCLUSIONS

This study evaluated the applicability of portable NIR spectroscopy, operated in both transmittance and reflectance modes, for the authentication of Brazilian coffees with four distinct GIs. Although both acquisition modes showed satisfactory performance during model calibration, reflectance consistently outperformed transmittance in the prediction of the test set, providing more stable and robust classification results.

The superior performance of DD‐SIMCA models developed from NIR reflectance spectra of ground coffee, compared with those based on aqueous extracts, can be attributed to the preservation of chemically and structurally rich information in the solid matrix, including lipids and other compounds that are poorly extracted into water. In contrast, spectra of aqueous extracts are largely dominated by water absorption, which attenuates class‐specific chemical signatures and increases intraclass variability, thereby adversely affecting class‐modeling approaches such as DD‐SIM*C*A.

Overall, these findings demonstrate that portable NIR spectroscopy, particularly when applied in reflectance mode and combined with DD‐SIMCA, represents a fast, non‐destructive, and reliable strategy for authenticating Brazilian coffees with protected GIs.

## FUNDING INFORMATION

Conselho Nacional de Desenvolvimento Científico e Tecnológico (CNPq) (grants #173330/2023‐1, #305628/2022‐4, #130939/2024‐2), Coordenação de Aperfeiçoamento de Pessoal de Nível Superior (CAPES) (grant #88887.821443/2023‐00), and Fundação de Amparo à Pesquisa do Estado de São Paulo (FAPESP) (grants #2023/00474‐4, #2021/06968‐3).

## Data Availability

Research data are not shared.
